# Trousseau’s Syndrome in Lung Cancer Patients: A Retrospective Study in a Japanese Community Hospital

**DOI:** 10.7759/cureus.68400

**Published:** 2024-09-01

**Authors:** Kohei Yoshimine, Kazunori Tobino, Yumi Obata, Shouta Sogabe, Kazuki Uchida, Yosuke Murakami, Ryuta Yamamoto

**Affiliations:** 1 Respiratory Medicine, Iizuka Hospital, Fukuoka, JPN

**Keywords:** prognosis, immune checkpoint inhibitor (ici), egfr mutation, thromboembolism, lung cancer, trousseau's syndrome

## Abstract

Trousseau's syndrome is a cancer-associated thromboembolism that significantly impacts patients' prognosis and quality of life (QOL). This study aimed to investigate the frequency, characteristics, and prognosis of Trousseau's syndrome in lung cancer patients at a Japanese community hospital and examine the effects of therapeutic agents on this condition. We retrospectively reviewed the electronic medical records of lung cancer patients diagnosed with thrombotic complications at the time of diagnosis in our department between August 2013 and April 2019. Patients' characteristics, thromboembolism sites, treatments, and prognosis were analyzed. Among 956 lung cancer patients, 19 (2%) had Trousseau's syndrome. The median age was 65 years, and adenocarcinoma was the most common histologic type (78.9%). The most common site of thromboembolism was the brain (84.2%). The median survival time was 84 days, and 52.6% of patients died within 90 days of diagnosis. Patients who survived longer than 90 days tended to have a higher frequency of non-adenocarcinoma histology, *EGFR *gene mutations, and therapeutic induction with immune checkpoint inhibitors (ICI). Trousseau's syndrome in lung cancer patients is associated with poor prognosis. Histologic type, *EGFR* mutation status, and treatment with ICI may influence the prognosis. Future larger-scale studies are needed to validate these potential prognostic factors and to develop personalized treatment strategies.

## Introduction

Since its first report by Armand Trousseau in 1865, Trousseau's syndrome has been defined in different ways by different researchers [1]. Currently, it seems reasonable to limit the use of Trousseau's syndrome to unexplained thrombotic events that precede or accompany the diagnosis of occult visceral malignancies [2]. Thrombotic events in patients with cancer are associated with poor prognosis and decreased quality of life (QOL), and in fact, median survival after cerebral infarction in Trousseau's syndrome has been reported to be 4.5 months [3]. Lung cancer is the most frequent malignancy associated with thrombosis, and the cumulative one-year incidence of thrombotic events in lung cancer has been reported to be 3% [4]. However, in recent years, the treatment of lung cancer has improved with the help of tyrosine kinase inhibitors (TKI) and immune checkpoint inhibitors (ICI). Therefore, the prognosis of Trousseau's syndrome in lung cancer patients is expected to have improved in recent years. In addition, the standard treatment for Trousseau's syndrome is heparin, but in recent years, direct oral anticoagulant (DOAC) has been reported to be effective for cancer-associated venous thromboembolism and is actually used for Trousseau's syndrome [5,6]. In this study, we investigated the prognosis of Trousseau's syndrome in lung cancer patients at our hospital in recent years and examined the differences in the effects of therapeutic agents on this condition.

## Materials and methods

Study design and patient selection

This retrospective study was approved by the Ethics Committee of the Iizuka Hospital (Number 22133), and the requirement for informed consent was waived due to the retrospective nature of the study. We included lung cancer patients diagnosed in our department between August 2013 and April 2019 who had thrombotic complications at the time of diagnosis. The histological type of lung cancer was not considered as an inclusion or exclusion criterion in this study. Lung cancer was diagnosed based on histopathological examination of biopsy specimens or cytological analysis of pleural effusion. Thrombotic complications were confirmed by appropriate imaging studies (CT, MRI, or ultrasonography) and included specific conditions such as cerebral infarction, renal infarction, splenic infarction, pulmonary embolism (PE), and superficial thrombophlebitis. Exclusion criteria were patients with only deep vein thrombosis (DVT), as DVT involves risk factors other than cancer; patients with concurrent malignancies other than lung cancer; and patients with incomplete medical records.

Data collection

In our study, two trained researchers independently extracted information from electronic medical records using a standardized data collection form. They gathered demographic data, including age and sex, as well as clinical data such as the Eastern Cooperative Oncology Group Performance Status Scale (ECOG-PS) and any comorbidities. Cancer-related data were also collected, focusing on the histology of lung cancer and its stage according to the 8th edition of the TNM classification. Additionally, the researchers recorded thrombosis-related information, including the location of thrombosis and its treatment. Furthermore, treatment and outcome data were documented, encompassing lung cancer treatments, survival status, and prognosis.

Treatment details

Anticoagulation or antiplatelet therapy for Trousseau's syndrome included DOAC, unfractionated heparin, warfarin, and aspirin. Lung cancer treatments were categorized as chemotherapy, TKI, and ICI.

Follow-up and outcome measures

Patients were followed from the date of lung cancer diagnosis until death or last follow-up, whichever came first. Follow-up assessments were conducted every four to eight weeks or as clinically indicated. The primary outcome was overall survival (OS), defined as the time from lung cancer diagnosis to death from any cause.

Statistical analysis

Descriptive statistics were used to summarize patient characteristics. Continuous variables were presented as median and range, while categorical variables were presented as frequencies and percentages. Fisher's exact test or Mann-Whitney U test was used to compare groups. For the purpose of evaluating the initial effects of lung cancer treatment and thrombosis management, we compared the group of patients who died early (within 90 days) with the group of patients who survived long-term (more than 90 days) to explore the differences between the two groups. A 90-day period was used as a benchmark. Kaplan-Meier survival curves were generated. All statistical analyses were performed using R Version 4.3.3 (The R Foundation for Statistical Computing, Vienna, Austria; https://www.r-project.org/). Statistical significance was set at p<0.05.

## Results

During this period, 956 patients with lung cancer were diagnosed at our hospital, 19 of whom were included in this study (2%, 14 male and five female) (Table 1).

**Table 1:** Patient characteristics Age is expressed as median (range). DOAC: direct oral anticoagulant, ECOG-PS: Eastern Cooperative Oncology Group Performance Status, NOS: not otherwise specified, NSCLC: non-small cell lung carcinoma, SCLC: small cell lung cancer

Characteristics (n = 19)	Results
Age, years old	65 (45-85)
Sex	
Male	14
Female	5
ECOG-PS	
PS 0	9
PS 1	8
PS 2	0
PS 3	1
PS 4	1
Comorbidity	
Diabetes	3
Hypertension	10
Dyslipidemia	5
Old cerebral infarction	3
Atrial fibrillation	2
Histopathology of lung cancer	
Adenocarcinoma	16
Squamous cell carcinoma	1
NSCLC, NOS	1
SCLC	1
Stage	
Stage III	1
Stage IV	15
Recurrence	3
Lesion of thromboembolism	
Brain	15
Kidney	3
Spleen	3
Pulmonary artery	2
Superficial thrombophlebitis	2
Anticoagulation or antiplatelet therapy	
None	6
Heparin	5
DOAC	4
Warfarin	3
Aspirin	1

**Figure 1:**
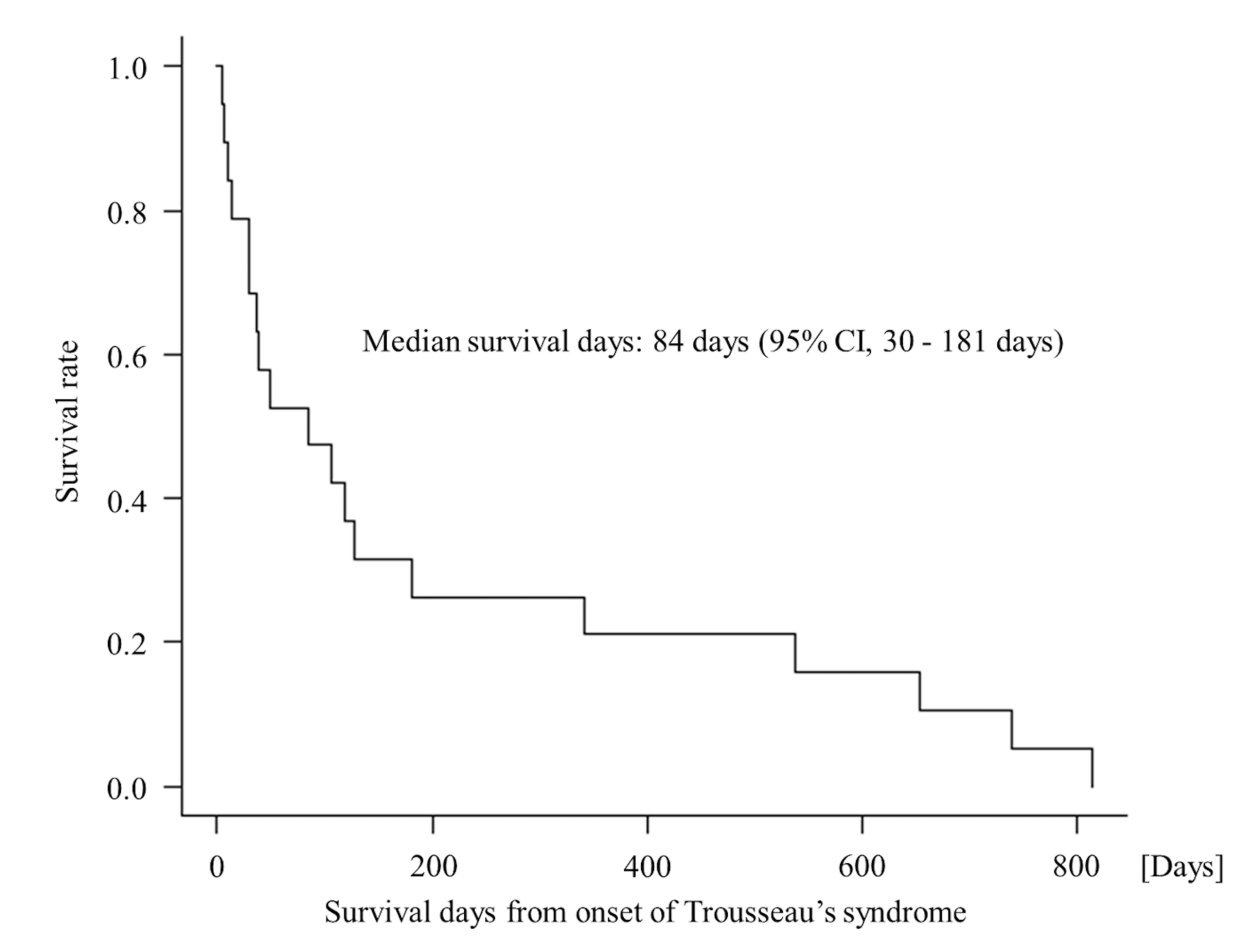
Survival outcomes following onset of Trousseau’s syndrome This figure presents the OS rates following the onset of Trousseau's syndrome, measured in days. The Kaplan-Meier survival curve depicts the proportion of patients surviving over time, with a median survival of 84 days (95% CI: 30-181 days). OS: overall survival

The median age was 65 years (range, 45-85 years), and 89.4% of the patients had a good ECOG-PS (0: 52.6%, and 1: 36.8%). Hypertension was the most frequent comorbidity (52.6%), followed by dyslipidemia (26.3%), previous cerebral infarction (26.3%), and diabetes mellitus (21.1%) with no cases of atrial fibrillation. Adenocarcinoma was the most common histologic type (78.9%), and stage IV was the most common clinical stage (78.9%). The most common site of thromboembolism was the brain (84.2%), followed by the kidneys (15.8%), the spleen (15.8%), and the pulmonary arteries (15.8%). Anticoagulation or antiplatelet therapy was started in 13 patients (68.4%) for the treatment of Trousseau's syndrome, and the details were as follows: DOAC, five patients; unfractionated heparin, four patients; warfarin, three patients; and aspirin, one patient. In addition, six patients (31.6%) did not receive treatment for Trousseau's syndrome. The median survival time for all patients was 84 days (95% CI, 30-181 days) (Figure 1), and 10 of 19 patients (52.6%) died within 90 days of diagnosis.

Comparing the group of patients who died within 90 days to the group of patients who survived longer than 90 days, there was a statistically significant difference in age (Table 2).

**Table 2:** Comparison of patients who died early (within 90 days) versus those who survived long-term (more than 90 days) DOAC: direct oral anticoagulant, ECOG-PS: Eastern Cooperative Oncology Group Performance Status, ICI: immune checkpoint inhibitor, NSCLC-NOS: non-small cell lung cancer, not otherwise specified, SCLC: small-cell lung cancer, TKI: tyrosine-kinase inhibitor

	Death within 90 days (n=10)	Survived more than 90 days (n=9)	P-value
Age, years old	72 (55-85)	63 (45-71)	0.05
Sex			1
Male	7	7	
Female	3	2	
ECOG-PS			1
PS 0	4	5	
PS 1	4	4	
PS 2	0	0	
PS 3	1	0	
PS 4	1	0	
Comorbidity			0.87
Diabetes	2	1	
Hypertension	7	3	
Dyslipidemia	3	2	
Old cerebral infarction	3	0	
Atrial fibrillation	2	0	
Histopathology of lung cancer			0.58
Adenocarcinoma	9	7	
Squamous cell carcinoma	0	1	
NSCLC-NOS	0	1	
SCLC	1	0	
Stage			1
Stage III	0	1	
Stage IV	8	7	
Recurrence	2	1	
Lesions of thromboembolism			0.16
Brain	7	8	
Kidney	0	3	
Spleen	2	1	
Pulmonary artery	2	0	
Superficial thrombophlebitis	0	2	
Anticoagulation or antiplatelet therapy: yes/no	5/5	8/1	0.14
Heparin	2	3	0.41
DOAC	2	2	
Warfarin	1	2	
Aspirin	0	1	
Driver gene mutation			0.14
EGFR gene	1	4	
None	9	5	
First-line treatment: yes/no	6/4	9/0	0.09
Cytotoxic chemotherapy	4	5	0.19
ICI	1	1	
EGFR-TKI	1	3	
IO treatment in all lines	1	2	0.58

There was also a trend toward differences between the two groups in the frequency of thromboembolic lesions, anticoagulant or antiplatelet therapy, frequency of EGFR mutations, and induction of first-line treatment. With regard to lesions of thromboembolism, there was no difference in the frequency of cerebral infarction, but renal infarction was only found in the survived longer than 90 days group and PE was only found in the died within 90 days group. For anticoagulation or antiplatelet therapy, five of 10 patients in the died within 90 days group did not receive it and eight of nine patients in the survived longer than 90 days group did. The frequency of EGFR gene mutations was three of nine patients in the survived longer than 90 days group, which was higher than in the died within 90 days group (one of 10 patients). Regarding the proportion of patients receiving first-line treatment, it was introduced in all patients in the survived longer than 90 days group, whereas it was introduced in a lower proportion (six of 10 patients) in the died within 90 days group.

## Discussion

In this study, we identified the frequency of lung cancer complicated by Trousseau's syndrome in Japanese community hospitals and characterized these patients. The frequency of 19 cases (2%) among 956 lung cancer patients was comparable to that reported previously [4]. The most salient characteristics of these patients were the high frequency of stage IV adenocarcinoma cases and the high prevalence of concomitant stroke. Survival in patients with Trousseau's syndrome has been reported to be short (median survival=4.5 months) and was even shorter in our cohort (median survival=84 days) [3].

Interestingly, some patients in our study were found to have a longer prognosis than previously reported. These patients tended to have a higher frequency of histologic types other than adenocarcinoma (squamous cell carcinoma, non-small cell lung cancer-not otherwise specified: NSCLC-NOS), a higher frequency of EGFR gene mutations (44.4%), and a higher frequency of therapeutic induction with ICI (22.2%). Although the sample size was small, these findings suggest that histologic type, EGFR mutation status, and treatment with ICI may influence the prognosis of patients with Trousseau's syndrome.

Trousseau's syndrome is the second most common cause of death among cancer patients, after cancer itself. It is characterized by cancer-related thrombosis, including DVT, PE, chronic disseminated intravascular coagulation syndrome (DIC), and arterial thrombosis [7]. The exact mechanism of Trousseau's syndrome is not fully understood, but factors such as circulating microparticles, secretion of procoagulant factors, altered platelet activity, and altered endothelial function may be involved [2].

In our study, NSCLC (94.7%), especially adenocarcinoma, was common, and many of these cases expressed EGFR mutations, similar to previous reports [8-10]. This suggests that the presence of mucin in adenocarcinoma may result in increased procoagulant secretion, and common driver gene mutations in NSCLC may be associated with VTE [9,10].

Although heparin has been reported to be effective in the treatment of Trousseau's syndrome, our study did not statistically confirm its efficacy [11,12]. This may be due to the small sample size or the potentially more significant effects of EGFR-TKIs and ICI on prognosis. Further research is required to clarify the optimal management of Trousseau's syndrome in the era of targeted therapies and immunotherapy.

The incidence of Trousseau's syndrome in the first half year after cancer diagnosis has been reported to be about two times higher in patients with distant metastases than in those without [13]. In our study, patients with adenocarcinoma of the lung and distant metastases were also common. The diagnosis of VTE has been associated with a higher risk of death within two years for lung cancer, as well as other cancer types [4,14-16].

It is important to acknowledge several limitations of our study. As a retrospective study conducted in a single Japanese community hospital with only 19 patients, our analysis is subject to inherent biases, including selection and information bias, and our statistical power is limited, which may have prevented us from detecting significant differences between groups and limits the generalizability of our findings. The retrospective nature also limits our ability to establish causal relationships and may introduce confounding factors. Furthermore, the limited number of patients treated with immunotherapy in our cohort may not reflect current clinical practice, as immunotherapy has become a mainstream treatment for lung cancer. The lack of a control group of lung cancer patients without Trousseau's syndrome prevents us from definitively attributing observed outcomes to the syndrome itself. Additionally, the inclusion of only patients with thrombotic complications at the time of lung cancer diagnosis may have led to an overestimation of the syndrome's prevalence and impact on prognosis. Finally, given that our study period spans from 2013 to 2019, changes in lung cancer treatment strategies over this time may have influenced outcomes and limited the applicability of our findings to current practice. Despite these limitations, our study provides valuable insights into the characteristics and outcomes of lung cancer patients with Trousseau's syndrome in a real-world setting. Future larger-scale, prospective, multi-center studies are needed to validate the potential prognostic factors identified in this study and to develop risk stratification models that can guide personalized treatment strategies for patients with lung cancer and Trousseau's syndrome.

## Conclusions

In this study, we found that the median survival of patients with Trousseau's syndrome in our hospital was shorter than previously reported in the literature. However, our findings revealed important nuances in patient outcomes. Notably, some patients, particularly those with EGFR mutations or those receiving ICI, demonstrated a longer prognosis. Based on these observations, we propose that when Trousseau's syndrome develops in lung cancer patients, continuous heparin should be administered, and genetic testing and PD-L1 staining should be performed to guide appropriate treatment as soon as possible. This approach aims to address both the thrombotic complications and the underlying malignancy, potentially improving patient outcomes. To advance our understanding and management of this condition, larger-scale, multi-center prospective studies are needed to validate the potential prognostic factors identified in our study. These future studies should aim to develop risk stratification models that can guide personalized treatment strategies for patients with lung cancer and Trousseau's syndrome. In conclusion, while our study provides valuable real-world insights into the characteristics and outcomes of lung cancer patients with Trousseau's syndrome, it also highlights the need for further research. By building on these findings, we can work toward improving the prognosis and QOL for this challenging patient population.
